# The Hospital. Nursing Mirror

**Published:** 1898-05-14

**Authors:** 


### "The Hospital" Nursing Mirror.

Being the Nursing Section of "The Hospital."

[Contributions for this Section of "The Hospital" should be addressed to the Editor, The Hospital, 28 & 29, Southampton Street, Strand, London, W.C., and should have the word "Nursing" plainly written in left-hand top corner of the envelope.]

### News from the Nursing World.

### INTERNATIONAL NURSING.

Once more the world stands witness to the barbarity of war. So slowly grows the nations' appeal from the axiom "Might is right" to that glorious decree "Right is might." Nurses have little to do with the ethics of war, but can they contemplate the banner of the Red Cross, unfurled so far in advance of international politics, without a thrill of joy and pride as they cry, "Behold the van of universal law!" Before 1864 the victor's spoils included the ambulance and hospital, whilst the wounded were left too often to die in the open. In that year, however, twelve European nations bound themselves to respect and protect those who carried medical and spiritual aid to the fallen. Thus, by virtue of the Convention of Geneva, the Red Cross proclaims safety in the very midst of the thunders and perils of the battlefield. Beneath this symbol succour has been taken to the wounded in many lands. France, Germany, Greece, Turkey, Spain, Cuba, Russia, and Rhodesia have shared its blessing; and now in America its votaries march in unbroken ranks to bind up the wounds inflicted by the sons of war, whether fighting under the Stars and Stripes or the Royal Eagle of Spain.

### THE KLONDYKE NURSES.

On April 16th the Rideau Hall, Ottawa, was the scene of a large farewell party to the four nurses about to leave for Klondyke, along with 200 Canadian troops. The outfits taken by the nurses, both of clothing and utensils and appliances for living and nursing requirements, were on view. Lord and Lady Aberdeen, who presided, Lieut.-Colonel Evans, who takes command of the contingent, and others addressed the assembly. Refreshments were provided, and the proceedings terminated about midnight.

### NORTHERN POLICE CONVALESCENT HOME AND ORPHANAGE.

St. George's College, Harrogate, has been secured for £10,000 to provide a convalescent home and orphanage for the police of the northern counties similar to the institutions already established at Reigate and Brighton. The secretary, Miss Catherine Gurney, is appealing to the public for funds to complete the purchase. The property consists of twelve acres of ground, and a handsome stone residence, built for a boys' school, which will accommodate 30 to 40 children and 30 adults.

### NURSING SMALL-POX.

When members of a committee come to loggerheads information of importance to the public leaks out. Mr. Galloway, a member of the Gateshead Town Council, has formulated a list of 19 allegations of mal-administration against the council. The point that concerns us is the assertion that three young nurses had the charge of two wards containing 30 patients suffering from small-pox, with the inevitable result that the work could only be imperfectly done. One delirious man was said to have been found dead on the floor, having thrown himself out of bed, dragging the clothes with him; and a mother saw fit to accompany her stricken children to the hospital in order to nurse them herself. Well might Mr. Galloway protest that these things ought not to be. When the public insists, and insists rightly, that infectious disease must be isolated, it is but just that the patients shall be assured of care and attention, and one of the first essentials is that there shall be an efficient staff of nurses of sufficient numbers to guarantee good nursing. We hope a full inquiry will be made into these allegations.

### NEW BEDROOMS AT THE NURSES' CLUB.

A pleasant surprise awaits members of the Victoria Commemoration Club. Three tiny but charming bed rooms have been added for their accommodation. These rooms will only be available for birds of passage, and the night's lodging, inclusive of bath and attendance, costs the modest sum of 2s. 6d.; breakfast that of 1s. The rooms are as artistic and comfortable as the rest of the domain under the control of the energetic secretary. It has been decided to discontinue the house lunch at a shilling, but members may order luncheon *à la carte*, which may make it cost either more or less, according to the nature of the meal required. A full luncheon, consisting of meat, vegetables, sweets, or cheese costs 1s. 6d. Everything is excellent of its kind, perfectly cooked, and daintily served.

### A FLOATING HOSPITAL.

In our number for April 30th we gave some account of the hospital ship prepared by the American Government, and staffed by Miss Enright and her band of Red Cross nurses. On May 4th the ship was ready to start. She has been rechristened the *Solace* (her name was the *Creole*), and she has been fitted with every appliance that the ingenuity of nursing science can suggest for the saving of life. Her hull is painted white, with a broad band of green, the object, of course, being to make her as conspicuous as possible, for the Red Cross protects her. Her burden is 3,800 tons, and her speed 17 knots. The operating theatre is in the forward saloon, and the wounded will be hoisted on board by a cable and tackle, and taken there at once if necessary. A dispensary and a bath-room have been fitted up immediately in the rear of the theatre. The main deck is fitted as wards, the forward part, containing 150 bunks, being arranged for the sailors, and the ward amidships on the same deck being reserved for the officers, whilst the after-part is used as a laundry. The steerage in the after-part of the ship has been turned into a comfortable convalescent-room. The *Solace* is accompanied by a couple of steam launches, which will visit the men-of-war and bring the wounded on board the hospital ship.

### THE WORKING FOLKS OF LEEDS CONVALESCENT HOME.

Drenching rain effectually checked any attempt at display on the occasion of opening the convalescent home at Horsforth, which has been acquired by the Leeds Workpeople's Hospital Fund. The home, which is solidly built, is in spic and span order, and will accommodate from thirty to forty guests. The chief entrance, giving access to a wide hall, is on the south, and is shielded by a large porch. To the east are the matron's rooms and domestic offices, and further away the roomy dining hall. On the west are the board-room and the men's recreation-room. An excellent view of the country is obtained from a verandah outside the latter. The two upper storeys are devoted to bedrooms, and the basement to store-rooms, larder, and a laundry. The house is heated throughout with hot water, and there are good baths, &c. The cost is wellnigh £5,000, £2,000 of which was taken out of last year's funds, £1,000 out of this year's funds, and £1,800 has been borrowed from a building society.

### AN AMATEUR ON NURSING.

A VAST amount of nonsense is written on the subject of nurses and nursing by people who have really no qualification for taking the platform on such matters. "A Lady" who contributes a letter to the *Birmingham Gazette* on "Hospital Nursing for Ladies," in the course of which the feeding of the nurses at one of the Birmingham Hospitals is complained of, shows plainly to the initiated that she knows not of what she writes. There is, as we have often contended, room for others besides the highly-educated in the nursing calling, but to lay down as an axiom that the daily work of the trained nurse, "the management of helpless patients, keeping wards clean and well ventilated, cooking for the sick, attending operations," are "duties in the performance of which culture would prove an obstacle and education no help" simply betokens ignorance of the subject under discussion. No doubt the food at many hospitals might be improved, and no one will deny that the hours are long, and that nurses are by no means overpaid for their work, but to give £25 a year as a maximum salary is contrary to fact. The "Lady" in question would do better if she left theorising on these matters to those who can speak upon them with knowledge and authority.

### BOLTON DISTRICT NURSING ASSOCIATION.

VALUABLE work is being done in a practical way by the Bolton District Nursing Association. Evidence of the appreciation in which it is held is shown in increased subscriptions. The Board of Guardians has raised its contribution from £50 to £100, the Cooperative Society now gives £50 instead of £30, the late Miss Walmsley bequeathed £500 to the society, and the Working Men's Hospital Saturday Committee have voted it 5 per cent. of the sums collected by them in the mills and workshops of Bolton. The income last year amounted to £933, and the expenditure was £874, thus leaving a balance which enables the committee to retain the services of the eighth nurse. This is the more satisfactory, as new streets are springing up in all directions, and the association must keep pace with the growing requirements of the place. Last year 1,306 cases were attended, and the medical practitioners withheld applications for nurses for fear of overtaxing their strength. The late Mayor had the greatest interest in the work, and regretted very much that the proposed appeal last year for extending it had to be delayed; consequently it is felt that his best memorial is to carry into effect his wishes on the subject. A committee has therefore been appointed to collect funds for the purpose, which will act in conjunction with the Jubilee Memorial Committee.

### NURSING IN SHANGHAI.

IT is proposed to establish in Shanghai, as a permanent memorial of the Diamond Jubilee, a nursing home and training institution, towards which a considerable number of subscriptions have been already received. The committee, in the first instance, approached the governors of the General Hospital with the view of achieving a co-operation between the hospital and the new institution. The governors do not consider, however, that any amalgamation can be effected, and the committee of the Jubilee Memorial have since applied to the Municipal Council with the idea of gaining the consent of the ratepayers to build the new home on public ground. It is estimated that a sum of Tls.25,000.00 must be raised in order to start the home upon a sound basis, and towards this the subscriptions at present collected and promised amount to Tls.20,000.00. The staff at first would consist of a matron and four trained nurses from England, with two probationers.

### ARGYLLSHIRE DISTRICT NURSING ASSOCIATION.

GREAT preparations are being made towards a bazaar which is to be held later in the year at Glasgow in aid of the District Nursing Association in Argyllshire. Various local committees are working hard to ensure its success, and the duties of secretary have been undertaken by Mr. R. C. Graham Campbell. The Duke of Argyll has promised his patronage, and is contributing some of his paintings to the sale.

### SHORT ITEMS.

OUR readers may be glad to know that there is a cycling school in Portman Rooms, Baker Street. A floral bicycle gymkhana, followed by a Cinderella dance, was held there on the 5th inst., and the proceeds devoted to the Cottage Hospital at Southwold. Hints as to first aid to the injured are given to pupils during their course of lessons.—A floral *fête* and bazaar, in aid of the Sturminster, Newton, and Hinton Nursing Fund, will be held in June at the residence of Mr. Fox-Pitt, the Manor House, Hinton St. Mary.—The National Society for the Prevention of Cruelty to Children is endeavouring to awaken the interest of the better class of children in those unfortunate little ones whose unhappy lot creates the necessity of protection. A guild called the Children's League of Pity has been formed, and at a recent meeting held in the Pump Room, Leamington, Mr. F. D. How, a son of the late Bishop of Wakefield, said that when he first took up the work there were 3,000 members, who in the course of a year collected £1,800. It is hoped that the membership will increase to 40,000, and that the money will enable the executive committee to extend the benefits of the N.S.P.C.C. to that third of the country not yet covered by its operations.—The Local Government Board has requested the reasons why the Guardians of Coventry have augmented their subscription to the Nursing Institution from £3 to £12. A statement will be made in due course to the effect that the money was spent to secure nursing attention for the out-paupers.—The following nurses passed the Medico-Psychological Examination held at the London County Asylum, Colney Hatch, N., on May 2nd: Nurses Dear, Greenaway, Gosborne, Heselton, Cherry, Cox, Dodd, Ross, Miles, Johnson, Warne, and Woodroffe.

### Lectures to Surgical Nurses.

By H. A. Latimer, M.D. (Dunelm), M.R.C.S. of Eng., L.S.A. of London, Consulting Surgeon, Swansea Hospital; President of the Swansea Medical Society; Lecturer and Examiner of the St. John Ambulance Association, &c.

XXXII.—ANTISEPTIC AND ASEPTIC SURGERY; DERIVATION OF THE TERMS—WHAT BACTERIA ARE: WHAT THEY EFFECT, AND FROM WHENCE THEY ARE DERIVED.

Now I will tell you of the modern modes of treating disease, which are known as the antiseptic and aseptic systems of surgery. Before describing to you these methods of treatment, and telling you on what great principles they are based, I had better explain what these terms actually mean; and I feel that in doing this I shall be giving you a good clue to what is to follow, for they are well-chosen words and are full of meaning. The first word means against, or opposed to putrefaction; the second, devoid of putrefaction. See, then, what is involved by them. In an antiseptic system of treating wounds a war has to be waged against poisons (which in this case mostly mean bacteria, or micro-organisms) which are already present in wounds, or which are attempting to invade them; in an aseptic system we are dealing with wounds, or matters connected with them, where there is an absence of those evil principles. The two systems have to be joined together in action, for however aseptic a wound is at its infliction, a war has to be waged against the entrance into it, or against their life after entrance, of these poisonous agents. The questions which concern us in considering the subject are these: From whence are bacteria derived? What are bacteria? What harm do they effect? How may we keep them at bay or destroy them if they are present? I will now attempt to give you satisfactory answers to these inquiries. Wherever dust is there we have bacteria, and as the air around us is full of dust you must regard it as being full of minute organisms which have their own purpose to fulfil as well as ourselves. Rivet this fact on your mind, and in your work wage a ceaseless war against dust and dirt, ever regarding these as agents for the dissemination of disease. There is no doubt but that dust has its good work to do as well as its bad; for the organic matter which it contains consists of beneficial organisms as well as of baneful ones. But, from the surgeon's point of view, it is matter in the wrong place, for its presence is fraught with disastrous results to health unless measures are adopted for destroying its harmful properties. The fact of dust being largely composed of micro-organisms is proved by examinations of it at different times under the field of the microscope, when it has been shown to contain these bodies in large numbers. For a long time past a number of scientific observers have been at work making these examinations, and the science of bacteriology—by which is meant the knowledge of the nature, varieties, and actions of bacteria, or micro-organisms—is being steadily extended. The varieties of these germs are infinite, and it is probable that the various diseases from which men, animals, and plants suffer all owe their continual life and reappearance from time to time, after periods of disappearance, to them. A great number of diseases have now been shown to be due to special kinds of germs; and, reasoning by analogy, it is likely that each one has its especial seed-bud to set it going. There is no such thing as "spontaneous generation," or the new birth, from nothing, of anything in life. All life is from the germ, and one kind of germ or another is constantly to be met with in the dust-laden air. Let me give you a simple illustration of this fact of the air as a carrier of germs. In the earlier experiments to prove this fact test tubes were filled with vegetable infusions which had been boiled to destroy any organisms therein contained; these tubes were placed alongside of each other, some of them plugged with cotton wool, and others of them with their mouths open to the atmosphere. The fluid in the protected tubes remained clear and pure for months; the fluid in the unprotected ones became turbid, and underwent fermentive changes in the course of a few hours. It has been shown over and over again that if a suitable medium for the life and development of micro-organisms is taken, and this material is sterilised (that is rendered barren), micro-organisms will appear and become developed on a portion of it which is exposed to the air, whilst another portion of it, which has been protected by having been covered up, will remain in its original state.

Another thing I want to point out about these minute forms of life is that they breed true; and that just as the seed of a daisy, if sown in suitable soil, and under favourable conditions, results in the springing up from the ground of a daisy-plant, and not of a convolvulus, or of a geranium, so the germs, let us say, of anthrax or of consumption, when received into the human body, under suitable conditions for their growth and development, result in the breaking out of those diseases in their new home. Perhaps you will ask me how such germs can get afield and be, as Shakespeare says of spirits:—

> "In the viewless winds,
> And blown with restless violence
> Round about the pendant world."

I shall reply that every bit of lint, or other fabric, soiled with a discharge from an infected person, or every such discharge itself which has been allowed to escape into the air, is loaded with these seeds of disease. Every consumptive person who spits into the road leaves there a fluid swarming with the germs of phthisis. After awhile the watery portion of this expectorated discharge evaporates, and the minute organisms which were formerly held together are liberated and are blown about as dust, to be inhaled by other people. And the same thing applies to all germs. This is how epidemics of disease suddenly appear in our midst.

A town will be free for a while from cases of scarlatina, and, suddenly, a number of persons suffering from that disease will be met with. Some condition of atmosphere has evidently favoured the development of the seed-bud of that complaint in suitable soils. Learn from these facts the importance which is attached to the destruction of discharges and "dressings" which have been soiled by discharges from invalids, and be ever mindful of the necessity of protecting wounds from the injurious influence of unfiltered air. And look upon your hands, and the instruments which you use in your work, as vehicles for the transmission of disease, unless they are constantly purified in one way or another.

The especial variety of germs which concern us now are those which set up suppuration and putrefactive fever. It would be useless refinement on my part to describe the forms of these micro-organisms; suffice it to say that such agents have been isolated and recognised, and that we now know that they are the direct producers of that state, and that they exist in our midst, but are kept from doing us an injury when the tissues of our bodies are in a healthy state. But if from any cause there is a lowering of the vitality of these structures the micro-organisms are enabled to penetrate them and to produce the evil which it is their purpose to effect. The bacteria which set up suppuration (that is the formation of matter) are minute dot-like bodies, sometimes clustered together, at other times arranged in chains. Pathologists tell us that they cause suppuration to occur partly by their own influence and partly by poisons which they secrete. It therefore becomes a matter of prime importance to prevent their reaching wounds, and to kill them if they have already got there, and we are taught how to do these things when we study the methods of procedure which are adopted in antiseptic surgery.

### Nursing in Paris Hospitals.

### B.—TRAINING LAY NURSES.

### II. THEORETICAL INSTRUCTION.

THE theoretical instruction at the four hospital nursing schools already established in Paris (those of Bicêtre, La Salpêtrière, La Pitié, and Lareboisière) is given in each case by the director of the hospital where the school is situated and by a staff of physicians. In the two last-named hospitals the number of medical lecturers is six, giving courses respectively (1) in anatomy, (2) physiology, (3) wound-dressing and minor surgery, (4) hygiene, (5) minor pharmacy, and (6) midwifery. At Bicêtre, Dr. Bonnaire combines the instruction in anatomy and physiology, while at La Salpêtrière the pharmaceutical instruction is given, not by a doctor, but by a chemist, M. Vion. At La Salpêtrière also, the course of instruction in midwifery is given, very appropriately, by a lady doctor, Madame Pilliet Edwards.

The theoretical instruction consists of 63 lessons, divided into seven courses, as follows: First course (hospital administration) of seven lessons—(1) Hospital schools; (2) Assistance Publique; (3) hospital history; (4) hospital organisation; (5) hospital routine; (6) duties of nurses; (7) criticisms of lay nursing. Second course (anatomy), in six lessons—(1) General anatomy; (2) skull; (3) rest of skeleton; (4) heart and organs; (5) nervous system; (6) respiratory organs. Third course (physiology), in six lessons—(1) Nuriture, &c.; (2) circulation; (3) respiration; (4) secretions, &c.; (5) organs of senses; (6) organic movements. Fourth course (dressings), of 18 lessons—(1) beds; (2) antiseptics; (3) splints; (4) topic remedies; (5) liquid topics; (6) internal topics; (7) injections; (8) salves; (9) mustard poultices and blisters; (10) bandages; (11) strait waistcoats, &c.; (12) leeching, &c.; (13) drownings, suffocation, &c.; (14) temperature hypodermic injections, vaccination and use of catheters; (15) auscultation and artificial alimentation; (16) eschar or scabs and parasites; (17) baths; (18) precautions against contagion. Fifth course (hygiene) in 12 lessons—(1) air; (2) light and heat; (3) electricity, winds, endemics, epidemics, disinfection; (4) salutary habitations; (5) heating and light; (6) clothing, bedding, &c.; (7) cosmetics, &c.; (8) food; (9) cooking; (10) special food; (11) adulterations; (12) drinks. Sixth course (lying-in) in four lessons—(1) hygiene and preparation for childbirth; (2) assistance at birth; (3) care of mothers after childbirth; (4) care of newborn infants. Of course the "lying-in" course is only for the minor emergency practice of the general hospitals. The complete midwifery course for La Maternité I will fully describe in a later article. Seventh course (pharmacy) in 10 lessons—(1) general pharmaceutical operations; (2) special pharmaceutical operations; (3) apozemes and poultices: (4) collutorium, collyrium, plaisters, sparadraps, diachylon, &c.; (5) emulsions, fomentations, fumigations, gargles, jellies, oils; (6) injections, liniments, lotions, pills; (7) salves, powders, &c.; (8) drops, sugars, suppositories, tinctures, alcoolatures, alcoolites; (9) decoctions and wines; (10) laxatives, purgatives, antiseptics. &c.

Each year in each of these seven courses (as well as in the course of practical instruction, which I shall next refer to), the students are given three subjects on which compositions must be handed in, and merit marks must be obtained according to a scale before a certificate is issued. The maximum required ranges from 50 per cent. in anatomy and 30 in physiology, to 60 per cent. in dressing, $60\frac{2}{3}$ per cent. in practical training, and 75 per cent. in administration, hygiene, pharmacy, and lying-in courses.

Every effort is made to induce all the nurses to strive for this certificate. Prizes for aptitude in studies are offered and distributed each year in great profusion, but as one of the speakers at an annual distribution of prizes remarked: "The certificate is everything, the prize *par excellence* of work, the reason for the study, the endorsement of aptitude for superior employment, the security for quick promotion, for the appointment of staff nurse or under matron. It is essential for all to have it, and to work with ardour to obtain it."

To take up the course of theoretical instruction the inducement is held out that not only in Paris but in the provinces. preference will be given to the certificated nurses. In 1894 a special table was compiled of all the grades of nurses with and without diplomas. The proportion may have been modified to a slight degree since in favour of the first class, but not to any serious extent. The table is as follows:—

| Grades. | | Total in Hospitals. |
|---|---|---|
| Male superintendents... ... | 4 | ... 234 |
| Matrons ... ... ... ... | 57 | |
| Assistant superintendents ... | 33 | ... 456 |
| Assistant matrons ... ... | 167 | |
| Male assistants... ... ... | 48 | ... 340 |
| Female assistants ... ... | 218 | |
| Male staff nurses ... ... | 50 | ... 423 |
| Female staff nurses ... ... | 180 | |
| Male dressers ... ... ... | 16 | ... 23 |
| Female dressers ... ... | 3 | |
| Porters ... ... .. ... | 5 | ... 23 |
| Male assistant nurses, &c. ... | 90 | ... 2,644 |
| Female assistant nurses, &c.... | 178 | |
| | 1,049 | 4,152 |

This table shows the gradual certificatising of all the upper grades, the largest percentage being in the middle grades, the highest being the older nurses who antedate the schools and are still retained, while the assistant nurses are not of sufficient length of service, as a rule, to have got the certificate. As to the upper grades, in the hospitals having schools M. Peyron has ordered those without certificates to attend the courses, but it would be hardly possible to enforce a similar order as to the other hospitals.

EDMUND R. SPEARMAN.

### Presentations.

AT a social gathering held by the nursing staff of the Union Infirmary, Sunderland, the superintendent nurse (Miss Pruett) presented a handsome travelling rug with straps to Nurse Bertha Parkes, as a mark of esteem, before her much-regretted departure to Canada. Miss Bertha Parkes was trained at the New Infirmary, Birmingham, and has held the post as charge nurse in the above infirmary for three years, during which time she has also obtained the certificate of the London Obstetrical Society.

MISS BELL has been presented with a cheque for £76 and a list of those subscribing to this testimonial of affection and appreciation. Miss Bell has for the last fourteen years been the lady superintendent of the Swansea and South Wales Institute, to which she has rendered valuable services. Lady Dillwyn Llewelyn, the chairman, was unfortunately absent, so the presentation was made by Miss Aubrey, who has been for many years the secretary.

### A Letter from Bombay.

### PLAGUE NURSING.

BY A SISTER ON DUTY.

SINCE writing my last letter from Poona, I have been transferred to Bombay, as the "plague" had decreased in the former place, and it was no longer necessary to have such a large staff of nurses there. I have been fortunate enough to have charge of the European Plague Hospital, working with a staff of four English sisters, four ayahs, and four ward-boys, three of the sisters taking day and one night duty. In our hospital, which, it may be remembered, was burnt down a little while ago, we have had an average of about 16 patients, "plague" fortunately not being very prevalent among Europeans. Most of these cases have been traced to infection from rats. A striking instance of this occurred when seven cases were brought from one house where rats were found dead in the flooring. Our work has been most interesting, though it was very hard at first owing to the severity of several of the cases.

The Government has been put to heavy expenses lately in the rebuilding and refurnishing of the hospital; however, we have now got pretty well all we want. At present all our patients are doing well; the two cases of the pneumonic form of "plague" having made good recoveries. It may here be interesting to note the different ways in which the buboes are treated. In this hospital, cold boracic compresses, changed frequently, are used; in one of the native hospitals the common treatment is the application of ice-bags; and in others, again, poultices are employed. And if I may be allowed to express an opinion, the application of "cold" appears to be most successful. The pneumonic form of "plague" is the most dreaded; it is often unaccompanied by a bubo, and the cases are always serious.

The European Plague Hospital is situated near the sea, and is built of wood, painted white, and thatched. We have two large wards, each capable of holding eight patients, as well as two smaller ones for convalescents. Cubicles are now being added for private cases. Our own quarters are built very much in the same way; we each have a bed-room and a tent as a dining-room. The nursing of European patients is much more after the style to which we have been accustomed, and it has been a very interesting change. Now, however, that the hot weather is rapidly approaching and the plague likely to decrease, rumours are afloat that we shall not be required much longer. We trust that there will be a final extinction of this dreadful disease, and that it will not crop up again, as it did last year.

The news of the fire and riots must now be quite a thing of the past. Everything is now much the same as usual, excepting that Europeans are more on their guard and that the hospitals are protected. Owing to the "plague measures" being more lax, there is not much fear of any further serious trouble. The experience of some of the nurses during the late fire and riots has been very exciting. Considering their extreme alarm at the time and the structure of our hospital, it is really astonishing that so many of the patients were saved, and they deserve great credit. One of the patients who was here during the fire and was with us until last week, described it to me. He is a tall man of over six feet, and the way he was carried out by the nurse was very clever.

We were all very much interested to see the pictures of our Poona camp in the *Illustrated London News*, especially as it must give our friends a very good idea of our surroundings.

The heat in Bombay is getting worse every day. It is impossible to describe the state of moisture in which we exist; but the cool sea breeze that blows sometimes of an evening is delightful. Bombay is a charming town, and the handsome buildings are most imposing. The band plays frequently in the evenings, and it is very enjoyable driving close to the sea front listening to the music. We nurses find Bombay very expensive as regards food, servants, and carriage hire. As I have explained before, in India driving is a luxury in which it is necessary to indulge frequently. It is therefore impossible to save much money.

The decrease in the plague rate is very cheering, but it is only natural that we should be looking forward to the time when we shall be returning home, leaving India, we trust, at last free from this disease. I think we have all enjoyed our nursing in India, everything having been made as comfortable as possible for the nurses. At present, although we are not as busy as we have been, nothing has been said about any of the nurses being sent back; and no doubt things will be left as they are until the "plague" shows definite signs of disappearing.

During the last two months, several of the nurses have been "inoculated." We all rather disliked the idea at first, as we heard various accounts of the pain and discomfort caused by it, but personally I did not mind it much. It certainly is unpleasant for a few days and has different effects on different people, but, judging from the good results of "inoculation," I think every nurse should certainly try it, and most of us have now been wise enough to do so.

### The Book World for Women and Nurses.

[We invite Correspondence, Criticism, Enquiries, and Notes on Books likely to interest Women and Nurses. Address, Editor, THE HOSPITAL (Nurses' Book World), 28 & 29, Southampton Street, Strand, London, W.C.]

NOTES ON PHARMACY AND DISPENSING FOR NURSES. By C. J. S. THOMPSON. The Burdett Series, No. 5. (London: The Scientific Press, 1898.) Price 1s.

What is quite clear is that to the ordinary nurse a great deal of the information contained in this little book will be quite superfluous. Still nurses are often placed in positions in which a great deal of it may be very useful, for although, as is truly said in the preface, the art of dispensing "can only be acquired by thorough practical instruction and experience," yet when a nurse is working at, say, a cottage hospital, or when she becomes a night sister, it may happen to her to have to handle drugs, and to give medicines and to do many things which may be done with much more comfort and satisfaction if she knows something about the materials she is dealing with, than if she is entirely ignorant of their properties and uses. The first chapter gives a description of the various preparations of drugs used in pharmacy. Then the various drugs are sorted out according to their actions into what we may call therapeutic groups. This portion of the book may seem to some a little dull, consisting as it of necessity does, to a large extent, of lists of the drugs contained in the several classes. When, however, we come to the chapters on the actual dispensing and compounding of medicines, things are much more interesting. The descriptions of the various processes are clear—we might almost say graphic—the rules are to the point, and the pages are full of useful information, which shows that they are the outcome of much practical experience. Altogether, we are very pleased with the book. We do not think that anyone could learn the art of dispensing from it, or, indeed, from any book; that, of course, is admitted in its pages. But we think that it will be a great help to those who without wishing to be pharmacists yet have some dispensing to do, as for example in the surgeries of general practitioners; while we have no doubt a careful perusal of it would smooth many rough places for juniors even among those w[illegible] take up pharmacy as their work in life.

### Mediæval Practice in the Care of the Sick.

### VII.—ON SOME OF THE OLD RECIPES HANDED DOWN TO US.

THE hands and head-piece of the nurse may be seen at work in the following recipes "against cancer": "Burn sulphur in a copper vessel, rub it to dust as small as thou may, sift through a cloth, mingle with old soap, and let the sulphur predominate, add a moderate quantity of virgin honey, see if it be too stiff, moisten with honey, and lay on with a mallow leaf."

And, moreover, in a 15th century manuscript we have preserved an actual representation of a nurse busy preparing a decoction for her patient. It is an illumination of the story of Tobit, who, sick and blind is shown, on a settle in the parlour; he has a bolster under his head and a covering over him, but he, nevertheless, looks most uncomfortable. Seated on a low footstool by the fire over which a cauldron is hung, is his wife; she is stirring some mixture, in another cauldron, with a spoon, according to directions given in the recipe book upon her knee, and her anxious countenance betrays her conscientious care and exactness. The illumination has been reproduced as a woodcut in Wright's book upon the "Manners and Sentiments" of our ancestors, and it is well worth study. We might almost imagine that Mrs. Tobit was preparing the following: "For mist of eyes . . . wring out juice of celandine, or of the blossoms of it and mingle with dumbledores honey, put it into a brazen vessel, then make it lukewarm cleverly on warm gledes or on ashes till it is done. That is a unique medicine for dimness of eyes."

Sick people in the Middle Ages must have suffered many things over and above their diseases, not the least being the uncomfortable want of either privacy or any approach to luxury in their surroundings. Our word bedstead is really a survival of the days when rooms were made with recesses, or alcoves down the sides, in which the family beds were placed. In Anglo-Saxon mansions there were often rooms with no beds at all, for in Alfred's vision of Bede "he had strewn for him *straw*," meant that he went to bed.

A sack filled with straw when required for use was laid upon a bench in the "bed-cofa," or crib, and an illuminated MS. shows us these beds formed in recesses; two people are represented lying in one with the curtains open, and below a woman is peeping through the curtain of the recess. The MS. is Alfred's version of Genesis; anyone who looks at the picture will cease to wonder that Saint Dunstan was able to go straight into King Edgar's hall to lecture him upon his marriage, and deliver his remonstrances, standing by the king and queen lying in their royal bed.

People went to bed naked in Saxon times, and "bed reafes," curtains, and sheets were valued possessions; an Anglo-Saxon lady left "two chests, with all the bedclothes that to one bed belong," by will to one of her children, and although we hear of rich cloth pillows being given as presents, the usual bolster and "pyles" were composed of straw.

Ladies' bowers were also the children's nurseries. Their noise, combined with what Devonshire people call the "smeech" of oil lamps, the smoke of the fires, and the hardness of the couches, must, one would imagine, have given wounded knights something to think of besides the bright eyes and the white hands of the ladies who tended them.

Perhaps the remembrance of such little drawbacks may account for the many stories told by the Romancers, of faithless knights, nursed back to life by gentle dames, who on their recovery rode off gaily with the ladies' hearts in their possession, to return no more to the scenes of their sickness.

The nursing performed by high-born damsels was of a very practical and thorough description; in the "Roman de la Violette," when Gerart, desperately wounded, is carried into the castle, a lady took him to a chamber, removed his armour, and put him to bed. Then she and her maidens examined his wounds, dressed them with salve, and the knight eventually recovered under the treatment. We know of a curious antiseptic used to prevent wounds from "fouling," "take briar, on which hips wax, that is dog-rose, chew the rind, and let it drop on the wound, and then it will not foul"; rather disagreeable for the nurse, and not so cleanly for the patient as a 1 in 20 solution of carbolic, one would think.

### The Meath Workhouse Attendants' Association.

THE Meath Workhouse Attendants' Association was founded in 1894 by the Countess of Meath, with the intention of introducing trained attendants into the wards in workhouses for the aged and infirm, epileptics and imbeciles, and infants.

Over sixty candidates have been carefully chosen, approved and accepted, as probationers of this association. These probationers receive their training in hospitals, homes, or infirmaries approved by the committee. Amongst these institutions are, the Royal Buckinghamshire Infirmary at Aylesbury; the Aubert Park Home, Highbury; the Birmingham Infirmary, the Birmingham Children's Hospital, Canterbury Hospital, Crampsall Infirmary, Manchester; St. Peter's Home, Kilburn; St. Lucy's Home, Gloucester; Memorial Hospital, Mildmay Park; Meath Home of Comfort; Moseley Hall Convalescent Hospital, St. Peter's, Woking; and the General Infirmary, Worcester. In 1896 the term of training for the probationers was extended, and now none of them receive less than one year's training. The majority remain for a longer period, and several are trained for two years.

Many more applications for these attendants have been received from boards of guardians than the association has been able to supply. More than thirty of them have obtained Poor Law appointments. Amongst those workhouses to which attendants have been appointed are, St. Pancras, Whitechapel, Islington, Poplar, Shoreditch, Blandford, Saffron Walden, Ouleston, Oxford, Ely, Isleworth, Chippenham, Scarborough, Hastings, North Walsham, Barton Regis, Cardiff, and Shepton Mallet. Between 30 and 40 probationers are now in training, and the reports received of them from the matrons of the institutions are very encouraging. The premiums for the most part have been paid by the Countess of Meath, but a few subscriptions have been received towards the expense. No salaries are given to probationers during their training. When they receive appointments under the Poor Law, the salaries are given by the Poor-Law Guardians, and vary from £20 to £30 a year. The committee endeavour to place such of their attendants as have had only one year's training in situations under fully qualified nurses, so that their training may be continued after the Poor-Law appointment is obtained. Country guardians have expressed their gratitude to the committee for the much-needed help which the work of this association has given them.

H. R. H. Princess Christian is the patron of the association. The Countess of Meath is the president. The vice-presidents are: Adeline (Duchess of Bedford), the Duchess of Rutland, Constance (Marchioness of Lothian), the Lady Bloomfield, the Lady Knightley, Mrs. Hugh Rathbone, Miss Home Cochrane, Mrs. Master, and Sir William H. Broadbent, Bart., M.D. Miss Mary E. Johnson, 18, Queen's Road, Richmond, Surrey, is the honorary treasurer. All information may be obtained by applying to Miss Lee, hon. secretary, at the office of the association, 51, Upper Baker Street, London, N.W. Office hours: Two to four daily.

### New Nurses' Home at Marylebone Infirmary.

The Guardians of the parish of St. Marylebone have lately added a new wing to the Nurses' Home which is attached to their infirmary at Notting Hill. This home now consists of 50 rooms, including a large lecture room, and comfortable sitting rooms, for the "Home Sister" and probationers. In addition to the probationers, the home accommodates the night nurses, a third of the house being entirely set apart for their use, so that quiet sleep is ensured for them during the day; the new wing was rendered necessary by the increase in the number of probationers, 24 being now in their first year of training.

The day nurses are accommodated in the administrative block, where are also situated the dining-rooms for "sisters" and nurses, and a pleasant recreation room, in which those who are musical can enjoy the use of a piano and small organ. Every nurse and probationer has her own separate bedroom.

A regular course of instruction by lecture and class is arranged for the year, the lectures and also clinical instruction being given by the medical superintendent, the class instruction by the "home sister"—this comprises physiology, anatomy, massage, and nursing subjects. Every six months written and *viva voce* examinations are held (the questions being set and the examinations conducted by a member of the staff of one of the London hospitals), for all probationers who have finished their course of instruction, the questions given in February, 1898, when the last examination was held, being as follows:—

1. Give a general description of the alimentary canal from beginning to end.
2. Describe the process of respiration and the changes produced in the blood, and in the ingoing and outgoing air by it.
3. Write a short description of the anatomy of the heart and of the functions performed by its several parts.
4. Write an account of the composition of normal urine and the commonest changes produced in it by disease.
5. How do you make beef-tea, peptonised milk, and make and administer nutrient enemata.
6. What would a nurse do—
   (*a*) To prevent bed sores.
   (*b*) In case of bleeding from the radial artery.
   (*c*) In case of sudden profuse hæmopytsis.
   (*d*) In bleeding from varicose veins.

In conclusion, it may be interesting to subjoin a list of appointments gained during the past year by nurses trained and certificated at St. Marylebone Infirmary: Assistant matron West Derby Infirmary, Liverpool; District Nurses to Rushden, Northants, and to the Paddington and Marylebone District Nursing Association; two nurses to the District Home, Bolton, Lancashire; night superintendent to the Hospital, Bury St. Edmunds; sister to the Thompson Memorial Home, Lisburn, Co. Antrim; two charge nurses to the Crumpsal Infirmary, Manchester; one charge-nurse to St. Pancras Infirmary, Highgate; two to the Northern Hospital, Winchmore Hill; two nursing sisters to the plague hospitals at Bombay and Poona; and one to the European Up-Country Nursing Association, India.

### Where to Go.

Wimborne House, 22, Arlington Street.—A conference, under the auspices of the Central Bureau for the Employment of Women, will be held here, by the kind permission of Lady Wimborne, on the 20th inst., at 3.30. Several noted ladies and gentlemen will address the assembly on questions relating to the employment of women. Tickets may be had from the Hon. Secretary of the Bureau, 60, Chancery Lane, W.C.

### The International Fancy Fair at Camden Town.

The International Fancy Fair is somewhat a pretentious title for the very pretty bazaar held in the Athenæum, Camden Road. The building was gay with bunting, and the hall was artistically decorated for the event, the different stands being canopied with white wooden fretwork draped with yellow, whilst the attendants added to the festive appearance by their fancy dresses. On the left of the entrance the succession of stalls were stocked by friends of the North-West London Hospital in Kentish Town, for the benefit of which the sale was arranged. At these stalls the attendants had arranged themselves to represent flowers—forget-me-not, daisies, poppies, roses, lilies. A beautiful flower booth, well placed across the foot of the platform, was liberally supplied with handsome plants and cut flowers by the generosity of the congregations of St. Paul's, Camden Square, and St. Luke's, Kentish Town. The congregation of St. Dominic's Priory, Haverstock Hill, St. Peter's, Belsize Park, and St. Barnabas, Kentish Town, each furnished stalls, whilst the tradesmen of the district united to give one stall. Swiss demoiselles, Italian peasants, gipsy girls, and maidens of the Austrian Tyrol were in waiting on this side of the hall, and flitted about plying a brisk trade in their various wares. The opening ceremony was performed by Mrs. Bischoffsheim, who was introduced by Mrs. Herring. Mrs. Bischoffsheim has not only given generously to the hospital herself, but she has interested others in its welfare, and on her representations the Baroness Hirsch has made a very handsome donation. The fair was open on May 9th, 10th, and 11th. The second day's opening was performed by Mrs. Herring, and the third by Lord Rathmore. It is hoped that a sum of not less than £800 has been made. The hospital needs £4,000 a year for maintenance, and only £800 of that is secured by subscriptions and endowment.

### Death in Our Ranks.

Miss Higgins (Sister E. Frances), whose death from plague on April 29th, at Kennedy Town Hospital, Hong Kong, we briefly announced last week, was trained at Guy's Hospital; she was afterwards a "sister" at St. Marylebone Infirmary, and left for Hong Kong in 1890, being one of the six sisters selected at that time to go out to the Government Civil Hospital at that place. Miss Higgins was presented with the medal for her services during the plague in 1894. Great sympathy is felt that she herself has fallen a victim to the disease so fatal amongst the Chinese, to numbers of whom she gave such unremitting care and attention. Miss Higgins was home on leave in 1896, returning to Hong Kong in December, the same year, and her untimely death is much regretted by all her friends at home and abroad. We also regret to announce the death of Sister Gertrude Ireland on May 5th of plague at Hong Kong. Although there is as yet no further information, it may be taken for granted that she had gone to Kennedy Town Hospital when Sister Frances was taken ill. She also was one of the first six sisters to go out to the colony in 1890, and nursed all through the epidemic of 1894. Trained at the London Hospital, she afterwards became night sister and then Sister "Blizzard" of the same hospital. Both these sisters had returned to Hong Kong for a further term of service after having been granted home leave. We extract the following from a letter which we have received from a lady who herself worked for five years as nurse in the Government Civil Hospital, and bore her share of the brunt of the epidemic of 1894: "Everyone connected with the Government Civil Hospital, and the whole colony, will sincerely mourn the loss of these two sisters, who, by their earnest zeal for duty and their kind, sympathetic dispositions, made themselves beloved by all."

### Post-Graduate Clinics for Nurses.

By a Trained Nurse.

### THE NURSING OF CHOREA.—II.

Dr. Weir Mitchell, who frequently ordered vigorous massage, instructed his nurses not only to deeply knead the muscles in these cases, but to make passive movements of the joints so as to control and counteract the more violent efforts on the part of the patient. His routine treatment of chorea cases consisted in putting the children on a diet of peptonised milk, from 48 to 56 ounces daily. Galvanism of the spine was applied daily for about ten minutes. Fowler's solution of arsenic was given hypodermically on alternate days; and given thus it is said to rarely cause nausea. Before giving the hypodermics the skin about to be pricked had to be well frozen with ice, and to prevent the formation of needle abscesses the needle must be carbolised both before and after use. Fowler's solution, with equal parts of glycerine, was always used for the hypodermics.

In chorea arsenic is sometimes "pushed," and very large doses given, so that the nurse must watch for symptoms of skin pigmentation, gastric pain, diarrhœa, vomiting, irritability of eyelids, &c., and report any one of these to the doctor in charge of the case. Control over bladder and motions will not be lost unless the chorea is accompanied by mental trouble. It must not be forgotten that most choreic children suffer from general feebleness, and that they easily tire both in body and mind. Not infrequently their heat needs keeping up by the use of hot water bottles, since the vitality is often much reduced, and the patient's extremities become very cold unless wrapped in cotton wool, or flannel, or kept warm by artificial heat.

The most important of all points in the care of the choreic has been left till last. For the good feeding of such a patient is the most essential step towards recovery of lost nerve power. The nurse frequently finds the patient an ill-nourished child, in whom want of appetite and disinclination for the exertion of taking food have led through previous months to a markedly insufficient dietary. So that the nurse must assume as her first duty the office of repairing the ill-nourished tissues, and making up for past deficiencies. She will often have to overcome, not only the patient's disinclination for food, but the fact that the attempt to take nourishment is frequently attended by much increased choreic movement. Swallowing, too, may be so difficult that much feeding appears at first sight to be an impossibility. It then becomes a question of skill and patience on the part of the nurse, and this she must be prepared to exercise, since so much of the success of the case will depend on the amount of nutriment given. In the very worst cases, and at the worst stages, the doctor may have to chloroform the patient while liquid nourishment is given through a soft tube passed down the gullet. This is resorted to only when the movements are so violently choreic that other methods of feeding prove impracticable. Nasal and rectal feeding are two other resources lying at the disposal of the nurse to supplement the amount of nourishment a choreic can take. A very satisfactory method of giving food in these cases is by the use of an infant's feeding bottle. And I would remind nurses to use those of the long-tube type, and not the boat-shaped bottle. I once saw a choreic bite involuntarily through the glass when this kind was used. The bottle with the long tube can be kept at a safe distance from the mouth.

I would like to emphasise the extreme necessity of good food for the choreic—of an easily-digested kind, for food which places a strain on the digestive powers is apt to increase the choreic movements. Cream, malt extract, custard, and whipped eggs should be given in generous quantity. In nursing such cases it may be necessary to put nourishment into the child's mouth by means of a medicine dropper—a tedious performance, but one which the nurse may not shrink from in time of need. Where swallowing is difficult, and the placing of a cup or spoon to the child's lips is accompanied by increased and violent movements and the clenching of the teeth, I have sometimes soaked a piece of cotton wool in a mixture of sweetened cream, yolk of egg, and malt extract or Mellin's food, and enclosing this in a piece of butter muslin on the principle of the sugar-rag so beloved of the working-class baby, have given it to the child to suck. In this way, especially if the nurse has the tact to leave the child to itself, a good deal of nourishment may be given. To look at the child in the act of feeding is enough in many cases to induce most violent jerkiness, partly through a self-conscious shyness, and partly owing to some degree of shame and terror on the part of a sensitive child at the small amount of self-control that he possesses.

I would like to remind nurses that the amount of muscular movement, and the nervous strain accompanying this, produces a great deal of exhaustion in the chorea patient, and this has to be counteracted by the food he takes; so long as the weakness and anæmia is not successfully combated, so long will the choreic condition last. Food, too, should be given at frequent and regular intervals. Want of food proves an exciting cause in all nervous disorders, and in none are the effects of too long abstention from nourishment more marked than in chorea. After food the patient usually quiets down and tends to sleep, and during sleep, unless in the worst cases, movements usually cease. Warm food should be chosen in preference to cold, as the former proves more soothing, and, if the diet be left more or less to the discretion of the nurse, she should avoid giving meat infusions and beef teas. A choreic does far better on generous quantities of milk, eggs, and farinacea.

The treatment adopted by some physicians in bad cases is to keep the patients under the influence of chloral so that they are always asleep, save when roused to take food.

Acute chorea is supposed to run a course of about six weeks, but it may go on for as many months, or even a year. When it proves fatal it is usually through heart complications or from mental degeneration. In all cases the nurse must watch for worms in the motions and should examine these, too, as a guide in the administration of food. If curds or any other evidences of undigested food make their appearance in the motions, it may be necessary to resort to peptonisation.

During convalescence over-tire and excitement must be avoided, and the child should be encouraged in the practice of rhythmical movements, such as those involved in dancing, skipping, and marching to music, or musical calisthenics.

### Appointments.

### MATRONS.

Preston and County of Lancaster Royal Infirmary.—Miss Kathleen Disney was elected Matron of this infirmary on May 2nd. She was trained at the London Hospital, and remained there for six years, serving as night sister for the last three. In 1894 she was appointed matron of the Boston Hospital, Boston, Lincolnshire, which post she relinquishes for her new work.

Dorset County Asylum.—Miss Emily K. Furnell has been appointed Head Nurse in succession to Miss Bird, who has been elected Matron of the new asylum for the Borough of Middlesbrough. Miss Furnell received her training at Dorchester.

St. Mary's Hospital, Manchester.—We regret that, by a printer's error, in our list of appointments last week the name of Miss Rose Emily Merrett, the new Assistant Matron of this hospital, was given as Miss Rose Smily.

### Everybody's Opinion.

[Correspondence on all subjects is invited, but we cannot in any way be responsible for the opinions expressed by our correspondents. No communication can be entertained if the name and address of the correspondent is not given, as a guarantee of good faith but not necessarily for publication, or unless one side of the paper only is written on.]

### PROS OVER THIRTY-FIVE.

"OLD MAID" writes: My experience may possibly be of use to inquirers about training over thirty-five years of age. At thirty-seven, I asked the late Mrs. Wardroper's advice on this subject, and she was very emphatic in her opinion that healthy active women over thirty-five made the very best of nurses. Acting on her advice, I trained for one year as a paying probationer at a Coventry Hospital, and have never been out of a good situation since. This is seventeen years ago, when one year's training was considered sufficient, but if three years had then been expected, I should certainly have agreed for it.

### THE DAUGHTERS OF THE KING.

"A KING'S DAUGHTER" writes: I read with much interest your notice in "The Mirror" of April 9th with reference to the King's Daughters' Society. But may I call your attention to the fact, that in its origin, this has never been an evangelising society of the Church Guild of any description; and the very great success of the work in America and Canada is due, no doubt, to the extreme simplicity and liberality of its professions. There women and children workers of all classes and denominations are included as members, and required only to try and live in accordance with the universal motive—"Look up, and not down," "Look in, and not out," and "Lend a hand to all," "In His Name"—the fulfilment of the same being left to individual character and choice.

### MENTAL NURSING.

"JESSIE M." writes: Relying on your evident interest in psychology, I send you my experience of mental nursing for the benefit of those who care to take up this absorbingly interesting work. Mental nursing is at present in a state of transition, and while our sisters of the hospitals and infirmaries have escaped the thraldom of "Sairey Gamp," mental nurses are not yet so fortunate. Mental nursing demands a rare combination of broad sympathy and rigid morality, for one of the distressing features of mental aberration is the moral obliquity of the patients and their inability to discern right from wrong, and a morbid dwelling upon erotic imagination which the true mental nurse must endeavour to counteract if she sets before her the example of the Great Physician, who not only healed the maimed and suffering bodies of His brethren, but also cast out the evil spirits that dominated the brain. It is those who have this high aim that realise the mighty power moral suasion has over the insane. People who hold that Christ was a great mesmeric healer may not be altogether wrong, for the unhappy sufferers from mental derangement are keenly susceptible to the mystic power of the moral nature and strength of those with whom they are brought into contact.

And is it not the most delicately sensitive minds that fall victims to mental disease? When we think of the great intellect of Dean Swift, with its haunting fear of inherited madness, of the gifted sister of the good and gentle Elia, of Cowper's clinging affectionate disposition, with its ever-recurring melancholy, we realise how these poor storm-tossed souls depended for guidance and happiness on those who surrounded them, and we cannot underrate the influence of the nurse over insane patients, especially in the present days of harmful over-pressure, when a perfect sanity is rarer than many dream of.

The care of incurable insanity is the darker side of the work, which also has its joys and triumphs. It is the privilege sometimes of the nurse to watch the gradual awakening of the returning intellect. How sweet and gentle some of those former maniacs prove when they emerge from the heavy cloud which has been over their poor tortured brains, and it is here that broad sympathies are needed; for a nurse must forget the idiosyncracies of her patients, and not hold them responsible for any act or speech of theirs, whilst insane. People are virtually changed during mania; many of them will say and do what in sane moments they would shudder to hear. I have not spoken of the social status of the mental nurse, for I believe that those who set overweening importance on this point seldom do any great work; but suffice it to say, that many mental nurses are women of education and culture, and everything is done for the comfort of all the nurses. Bright, airy bed-rooms, a cheerful sitting-room, and a library of the latest books and magazines; while the lectures, medical and surgical, are not one whit behind the very excellent hospital in which I am now training as a nurse.

### THE FIRE AT MODI KHANA.

"A LOVER OF JUSTICE" writes: The description of the recent fire at Modi Khana Plague Hospital, which appeared in the *Nursing Record*, was slightly erroneous and also gave a very wrong impression of the behaviour of the Parsis on that occasion. Unfortunately, several members of the community were presented with a copy of the *Nursing Record*, and naturally much resent the way in which their conduct was represented. In the first place, may I point out that the fire did not begin at the Native Plague Hospital, but at the European one, which is near, and nearly all the inmates of the native hospital were carried out of the wards before the flames actually reached them. In this respect the Parsi wards were most favourably placed, as they were on the farther side of the compound, and as each Parsi patient has a friend or relative constantly with him, there was little difficulty in getting them quickly out of danger's way. That the said relations and friends deserted them at this critical time is most untrue, and anyone who has any knowledge of the Parsis cannot fail to be struck by the untiring devotion and attention they pay to their sick relatives; indeed, it is against their creed ever to have them left unattended by one of their own caste. That the English sisters behaved with courage is only what we should have expected, and we were not disappointed, but as the other nurses present did so too, also the native assistants and orderlies, it is unfortunate that some should have been singled out for special praise, when others, who were equally courageous, were not even mentioned. Trusting that you will insert this correction of the exaggerated account of the fire that appeared before in the columns of the *Nursing Record*.

*** We have also received a letter from a "Parsee" upon this subject, but as it is unaccompanied by name and address we are not able to print it. The writer, however, has our sympathy.—ED. *T. H.*

### Grand Concert at Stafford House.

A GRAND afternoon concert was given on the 11th inst. at Stafford House, by the kind permission of the Duke and Duchess of Sutherland, in aid of the Special Appeal Fund of Charing Cross Hospital. The magnificent hall and staircase were thronged with guests, amongst whom were the Duchess of York. Her Royal Highness, who is still in deep mourning, was received on behalf of the committee by the Duchess of Portland, the Countess of Chesterfield, the Countess of Dudley, and Mr. George Drummond, the treasurer of the hospital. A number of well known artists generously gave their services. Miss Cissy Loftus' clever impersonations were much applauded. Master H. Vernon Warner, a prodigy of ten, played three pianoforte solos with wonderful facility. Mr. Maurice Farkoa's three songs were well received, especially "Charity," by Cicely McDonell, the alteration of a word here and there having made it most applicable to the occasion. The Croatian band, in their picturesque costume of red, blue, and gold, played selections of their national music at intervals during a very lengthy programme. Amongst those who then generously placed their talents at the service of the hospital, were Miss Clara Butt, Mrs. Brown Potter, Miss Letty Lind, Miss Louie Freear, Miss Marie Tempest, Mr. Ley Vernon, Mons. Johannes Wolff, Mr. Norman Salmond, Mr. H. Lane Wilson, &c.

Programmes, which were very nicely illustrated and contained the words, were sold by nurses from the hospital, in uniform. It is estimated that upwards of five hundred persons were present. Refreshments were provided gratuitously by Messrs. Gunter, for the benefit of the fund, and tickets for the tea room were freely sold at a shilling apiece.

### For Reading to the Sick.

"ALL of you be subject one to another, and be clothed with humility."

**Verses.**

If humble, next of thy Humility beware!
And lest thou should'st grow proud of such a grace, have care! —*Trench.*

Well doing bringeth pride;—this constant thought
Humility—that thy best done is nought. —*Bridges.*

Let the will kneel within thy haughty heart,
For benefits and meek submission tame
The fiercest and the mightiest. —*Shelley.*

Mortal! if life smile on thee, and thou find
All to thy mind,
Think, *Who* did once from Heaven to Hell descend
Thee to befriend!
So shalt thou dare forego, at His dear call,
Thy best—thine All. —*Keble.*

In obedience and humility,
Waiting on God's Hand, not forestalling it,
Seek not to snatch presumptuously the palm
By self-election; poison not thy wine
With bitter herbs if He has made it sweet;
Nor rob God's treasuries because the key
Is easy to be turned by mortal hands.

The gifts of Birth, Death, Genius, Suffering,
Are all for His hand only to bestow,—
Receive thy portion and be satisfied!
Who crowns himself a king is not the more
Royal; nor he who mars himself with stripes
The more partaker of the Cross of Christ.
—*H. Hamilton King.*

Hide me, oh Father, till the hour of death,
In lowly, silent hamlet ministry:
The rough and hard and homely task for me,
Nor angel-flights' mid flattery's poison breath!
He deigned forget His own Eternal Being. . . .
He loved and served and toiled, the end foreseeing—
Say were such lot too low for such as I? —*Morgan.*

If that in sight of God is great,
Which counts itself for small,
We by that law Humility
The chiefest grace must call;
Which being such, not knows itself
To be a Grace at all. —*Trench.*

**Reading.**

Humility is the most excellent natural cure for anger in the world; for he that by daily considering his own infirmities and failings makes the error of his neighbour to be his own case, and remembers that he daily needs God's pardon and his brother's charity, will not be apt to rage at the levities or misfortunes or indiscretions of another; greater than which he considers that he is very frequently and more inexcusably guilty of.—*Bishop Jeremy Taylor.*

Be extremely small and lowly in your own eyes, soft and yielding as a dove, loving lowliness, and cultivating it faithfully. Make good use of every opportunity for so doing. Do not be quick of speech, rather let your words be slow, humble, and gentle, and let your modest, thoughtful silence be eloquent. Bear with your neighbour, and be ever ready to make excuses for him. Do not philosophise over the contradictions which beset you: do not dwell upon them, but strive to see God in all things without exception, and acquiesce in His will with absolute submission.—*S. Francis de Sales.*

### Minor Appointments.

UNION WORKHOUSE, MARLBOROUGH.—Mrs. Mary Elizabeth Hesford has been appointed Matron of this workhouse. She was trained at the Union Infirmary, Salford, and has since been head nurse of the Union Infirmary, Newcastle-under-Lyme. Her husband was appointed Master of the Marlborough Workhouse at the same time.

SUTTON, CARLTON, CROMWELL, AND NORWELL.—Miss Kate Bruce Biggins was appointed Senior Nurse of this district on February 1st. Miss Biggins was probationer, and afterwards nurse for several years, at the Stockport Infirmary. She has since been engaged in private and district nursing. Her last appointment was on Lord Iveagh's estate, Elveden, Thetford, Norfolk.

### Notes and Queries.

**The contents of the Editor's Letter-box have now reached such unwieldy proportions that it has become necessary to establish a hard and fast rule regarding Answers to Correspondents. In future, all questions requiring replies will continue to be answered in this column without any fee. If an answer is required by letter, a fee of half-a-crown must be enclosed with the note containing the enquiry. We are always pleased to help our numerous correspondents to the fullest extent, and we can trust them to sympathise in the overwhelming amount of writing which makes the new rules a necessity. Every communication must be accompanied by the writer's name and address, otherwise it will receive no attention.**

*Bloemfontein.*

(61) Could you tell me whether there are any hospitals at Bloemfontein at which I might apply for work? Is the climate likely to be favourable to a lady of 30, with acknowledged "chronic bronchitis" and an unfavourable tubercular history on both sides of her family?—*Maud.*

Bloemfontein stands in a high and healthy district. But it is a small town, and nursing in the out districts is hardly work for a delicate woman. You are in the very centre of expert knowledge on the subject. Why not get a definite opinion on your case and its treatment? We are sure that one or other of the physicians of your hospital would advise you well.

*Training Men Nurses.*

(62) Can anyone tell a young gentleman with some knowledge of nursing where he can be trained in general nursing? An institution near Manchester and giving a certificate preferred.—*Opus et Pretiosus.*

There is no institution in Britain where a man may receive training in general nursing excepting the National Hospital for the Paralysed and Epileptic, Queen's Square, Bloomsbury.

*Training.*

(63) I cannot afford to pay a premium nor to give much time. Kindly tell me how I can best gain some hospital training in nursing, especially district nursing. I am 27, strong, and have had private experience.—*A. S.*

Write to Miss Peter, 1, St. Katherine Royal Hospital, Regent's Park, N.W. We strongly advise "A. S." to take a three-year course. Then she will receive her certificate, have nothing to pay by way of premium, and begin to draw a salary in her second year.

*Jamaica.*

(64) Can I obtain a three-year certificate in Jamaica? Or should I train first and go abroad later?—*Weathercock.*

Train in England first most certainly. Jamaica has two hospitals, but there seem no facilities for training nurses in either. The climate in parts is not very healthy, and at times yellow fever is prevalent.

*How to Become a Nurse.*

(65) Please tell me how to become a nurse? (2) Should I have to study before becoming a probationer?—*Nellie.*

Apply to the matron of which ever hospital or infirmary you prefer. She will give you an interview, and if you are suitable will tell you exactly what to do. (2) You seem well enough educated from your letter.

*District Midwife.*

(66) I am advised by my committee, on the termination of my engagement as district nurse, to remain in the neighbourhood as midwife and take private work. As I am dependent on my own exertions I should like your advice upon the subject.—*Perplexed.*

If "Perplexed" has already a connection in the neighbourhood and the good word of her committee she ought to have no difficulty in succeeding in private work, but much depends upon her repute among the medical men in the neighbourhood. If she does not succeed after a fair trial she can always return to institutional work.

*Who Ought to Disinfect?*

(67) Could you inform me if in district nursing of infectious cases it is the duty of the sanitary authorities to disinfect the rooms or of the district nurse?—*F. C. G.*

The nurse will, of course, notify any infectious cases to the sanitary authorities, if this has not already been done. She will also do her best to prevent infection spreading by taking and teaching others to take necessary precautions. This is the natural duty of those who possess special knowledge. Legally, the sanitary authorities ought to disinfect, but as they are not all perfect a nurse will often have to make up for their imperfections.

